# Improving precise point positioning performance based on Prophet model

**DOI:** 10.1371/journal.pone.0245561

**Published:** 2021-01-19

**Authors:** Shujian Liao, Chenbo Yang, Dengao Li

**Affiliations:** 1 College of Information and Computer, Taiyuan University of Technology, Jinzhong, China; 2 Shanxi Engineering Technology Research Center for Spatial Information Network, Jinzhong, China; 3 College of Data Science, Taiyuan University of Technology, Jinzhong, China; University of Shanghai for Science and Technology, CHINA

## Abstract

Precision point positioning (PPP) is widely used in maritime navigation and other scenarios because it does not require a reference station. In PPP, the satellite clock bias (SCB) cannot be eliminated by differential, thus leading to an increase in positioning error. The prediction accuracy of SCB has become one of the key factors restricting positioning accuracy. Although International GNSS Service (IGS) provides the ultra-rapid ephemeris prediction part (IGU-P), its quality and real-time performance can not meet the practical application. In order to improve the accuracy of PPP, this paper proposes to use the Prophet model to predict SCB. Specifically, SCB sequence is read from the observation part in the ultra-rapid ephemeris (IGU-O) released by IGS. Next, the SCB sequence between adjacent epochs are subtracted to obtain the corresponding SCB single difference sequence. Then using the Prophet model to predict SCB single difference sequence. Finally, the prediction result is substituted into the PPP positioning observation equation to obtain the positioning result. This paper uses the final ephemeris (IGF) published by IGS as a benchmark and compares the experimental results with IGU-P. For the selected four satellites, compared with the results of the IGU-P, the accuracy of SCB prediction of the model in this paper is improved by about 50.3%, 61.7%, 60.4%, and 48.8%. In terms of PPP positioning results, we use Real-time kinematic (RTK) measurements as a benchmark in this paper. Positioning accuracy has increased by 26%, 35%, and 19% in the N, E, and U directions, respectively. The results show that the Prophet model can improve the performance of PPP.

## 1 Introduction

Since the beginning of the 21st century, location-based service applications had become more widespread. With the continuous development of RTK, the positioning accuracy of GNSS has reached centimeter level [1]. This makes automated vehicles (AVs) more and more realistic [2]. Positioning accuracy determines the level of development of autonomous vehicles. However, there are many problems with the practical application of RTK. Firstly, an effective reference station cannot be established in the wild and the vast sea area. Secondly, the positioning accuracy will decrease rapidly with the increase of the baseline length [3].

Therefore, Zumbeger of the Jet Propulsion Laboratory proposed PPP in 1997. PPP had become one of the research hotspots in the field of satellite navigation [4]. IGS can provide precise ephemeris and SCB sequence. PPP can eliminate ionospheric errors through dual-frequency combined observations and eliminate tropospheric errors through modeling, which makes PPP a substitute for RTK in many scenarios [5]. Although IGS provides SCB sequence, its lag time is too long to meet general positioning requirements [6]. Therefore, it is necessary to obtain high-precision real SCB sequence. It will make PPP get better positioning results, and contribute to the development of automated vehicles and other location-based service applications.

Therefore, scholars have done a lot of research on how to obtain high-precision real-time SCB sequence. These methods are suitable for short-term, medium-term, and long-term predicting of SCB of navigation satellites under different conditions. But they all have their own applicable scopes and limitations. The Quadratic Polynomial Model (QPM) was first proposed to solve the SCB. The QPM uses time as a variable. Coefficients are determined by historical data. Then the model with known coefficients can predicts SCB sequence. Its advantage is that it can be fully fitted using longer time historical data. The disadvantage is that the predicted bias will continue to increase with time. It is suitable for short-term predict of SCB sequence [7]. Grey Model (GM(1,1)) was also one of the methods of SCB predict. GM(1,1) can use less historical data for modeling and predicting. But the model requires that data to change exponentially, which limits its use range. It is suitable for the long-term prediction of SCB [8]. Least Square Support Vector Machines (LS-SVM) can solve the problems of small samples and nonlinearity. But it is easy to fall into local minimization. It is suitable for mid-to-long-term predicting of SCB [9]. Auto-Regressive (AR(p)) is a hotspot model to study SCB in recent years. But the AR(p) model is only suitable for the prediction of stationary SCB sequence [10, 11]; Optimization-based methods are widely used in the field of prediction. It mainly obtains the extreme value of the target through various constraints. This method has high accuracy, but the calculation is complex and lacks real-time performance [12, 13]; Wavelet Neural Network (WNN) combines the advantages of artificial neural network and wavelet analysis in one method. It has fast network convergence speed, frequency local analysis, and other advantages. But as the input dimension of the network increases, the samples trained by the network increase exponentially. At the same time, the number of nodes in the hidden layer is difficult to determine [14, 15]. In terms of current research status, WNN is a representative method with higher accuracy.

In this paper, Prophet model was proposed to predict SCB to solve the problem in the existing methods. Firstly, uses IGU-O with a sampling interval of 15 minutes provided by the IGS station to predict SCB. Then, subtract the SCB sequence between adjacent epochs to obtain the SCB single difference sequence. Next, the Prophet model is used to predict SCB single difference sequence. Finally, the prediction result is substituted into the PPP positioning observation equation to obtain the positioning result. By comparing different prediction methods, the effectiveness of the method in this paper is verified.

The structure of this paper is as follows: the first chapter introduces the research significance of this paper and summarizes the existing SCB prediction methods; the second chapter introduces the algorithm and model used in this paper; the third chapter introduces the experimental arrangement and results; the fourth chapter summarizes this paper.

## 2 Methods

### 2.1 PPP mathematical model

The standard observation equations for satellite navigation systems are:

$$P=\rho +c(\partial_{t}-\partial_{T})+I+T+d_{mul}+d_{tide}+d_{res}+\varepsilon$$

$$L=\rho +c(\partial_{t}-\partial_{T})+\lambda N-I+T+d_{mul}+d_{tide}+d_{res}+\varepsilon$$ (1)

Where *P* is the pseudorange observation value; *L* is carrier phase observation. The parameter *ρ* is the geometric distance between the satellite position at the time of satellite signal transmission and the receiver position at the time of reception. *c* is the speed of light in vacuum, *c* = 2.99792458×10^8^m/s. *λ* is the wavelength of the corresponding frequency. *∂*_*T*_ denotes the receiver clock bias. *∂*_*t*_ denotes SCB. *T* is the tropospheric delay error. *I* is the ionospheric delay error. *d*_*mul*_ is the error caused by the multipath effect. *d*_*tide*_ is the error caused by the tidal effect. *d*_*res*_ is the error caused by the relativistic effect. *N* is the ambiguity of the corresponding frequency and phase observations. *ε* is the effect of observation noise and errors that are not modeled.

The PPP uses the phase and pseudorange observations at the L1 and L2 frequencies for ionospheric-free combination to eliminate the effects of ionospheric delay. The observation equation is:

$$P_{IF}=\rho +c(\partial_{t}-\partial_{T})+Md_{trop}+\varepsilon_{IF}$$

$$L_{IF}=\rho +c(\partial_{t}-\partial_{T})+N_{IF}+Md_{trop}+\varepsilon_{IF}$$ (2)

Where *P*_*IF*_ is the pseudorange observation of the ionosphere-free. *L*_*IF*_ is the phase observation of the ionosphere-free. The parameter *ρ* is the geometric distance from the receiver to the satellite. *∂*_*T*_ denotes the receiver clock bias. *∂*_*t*_ denotes SCB. The parameters *d*_*trop*_ is the zenith tropospheric delay. *M* is its mapping function. *N*_*IF*_ is the overall ambiguity of the ionosphere-free. *ε*_*IF*_ is the effect of observation noise and errors that are not modeled [16, 17].

It can be seen from Eq (2) that for the PPP model, except for the errors that cannot be eliminated, SCB has the greatest impact on the positioning accuracy. Therefore, the prediction accuracy of SCB has become one of the key factors restricting the positioning accuracy.

### 2.2 Prophet model

The Prophet model is a data prediction tool for the time series of Facebook. The model can not only handle the situation where there are some outliers in the time series, but also deal with the case of some missing values. It can also predict the future trend of the time series almost automatically. The Prophet model is based on time series decomposition and machine learning fitting, so it can get the results that need to be predicted in a faster time. On the whole, general time series prediction or data analysis can try to use this algorithm to predict the trend of future time series [18].

The essence of the Prophet model is an additive model, the basic form is:

$$y_{t}=g_{t}+s_{t}+h_{t}+\varepsilon_{t}$$ (3)

Where *g*_*t*_ is a trend term used to analyze acyclic changes in the time series. The parameter *s*_*t*_ is the periodic term used to analyze the periodic change in the time series. *h*_*t*_ is a holiday term used to analyze points in the time series that cannot be described by the model. *ε*_*t*_ is the error term and follows the normal distribution.

#### 2.2.1 Trend item

The growth of trend items is similar to the growth of the number of races in the ecosystem. It reaches a saturation value after experiencing nonlinear growth. This type of growth often uses a logistic growth model. The basic form is as follows:

$$g_{t}=\frac{C}{\text{1}+e^{-k(t-m)}}$$ (4)

Where *C* is the saturation value. The parameter *k* is the growth rate. *m* is the offset parameter. The Prophet model has improved it, setting both the saturation value *C* and the growth rate *k* to change with time. Set several turning points in the time series *s*_*j*_, *j* = 1,…,*s*, the growth rate will change at these turning points. Let *δ*_*j*_ denote the amount of change at time *t*_*j*_, construct a vector *a*(*t*)∈{0,1}^*s*^ as follows:

$$a_{j}(t)=\{\begin{array}{l} \text{1}\;t\geq s^{j}\\ 0\;\text{otherwise} \end{array}$$ (5)

The expression of the growth rate at time t is:

$$t=k+a(t^{T})\delta$$ (6)

When the growth rate changes, the offset parameter m should also be adjusted accordingly to connect the end of the time segment. The amount of offset adjustment at the turning point *j* is as follows:

$$\gamma_{j}=(s_{j}-m-\sum_{l<j}\gamma_{l})(1-\frac{k+\sum_{l<j}\delta l}{k+\sum_{l<j}\delta l})$$ (7)

The final trend item is:

$$g_{t}=\frac{C(t)}{\text{1}+e^{\left(-(k+a{\left(t\right)}^{T})\delta )(t-(m+a{\left(t\right)}^{T})\gamma \right)}}$$ (8)

#### 2.2.2 Periodic items

The periodic term is simulated by Fourier series:

$$s_{t}=\sum_{n=\text{1}}^{N}\left(a_{n}\text{cos}(\frac{\text{2}\pi nt}{p})+b_{n}\text{sin}(\frac{\text{2}\pi nt}{p})\right)$$ (9)

Where *p* is the period, when *p* = 1, the period is depicted in days. *a*_*n*_ and *b*_*n*_ is the parameter to be set. From the nature of the Fourier series, *n* can describe the changing periodic pattern.

#### 2.2.3 Random items

Similar to the periodic item processing method, a matrix of regression elements is generated:

$$z(t)=[1(t\in D_{1}),\ldots ,1(t\in D_{L})]$$ (10)

$$h_{t}=z(t)\kappa$$ (11)

Where *κ* is normally distributed.

The general prediction model method takes SCB as a whole, but the overall modeling effect is not ideal. We use the Prophet model to divide SCB into four parts, the trend item, the period item, the random item, and the error item in this paper, and finally obtains the SCB prediction sequence.

## 3 Results and discussion

IGS provides high-precision and continuous products for GNSS users, including satellite orbit, satellite clock, IGS station coordinates and speed, IGS station receiver clock, geocentric parameters, troposphere, and ionosphere products. High precision and continuity provide the basis for high-precision navigation and positioning for GNSS users [19]. There are four types of SCB products: broadcast ephemeris clock, ultra-fast ephemeris clock (IGU), rapid ephemeris clock (IGR), and final ephemeris clock (IGF). Its accuracy is inversely proportional to the delay, as shown in Table 1.

**Table 1:** SCB products provided by IGS.

Type	Accuracy	Latency	Sample Interval
Broadcast	5 ns RMS	Real-time	daily
Ultra-Rapid (predicted half)	3 ns RMS	Real-time	15 min
Ultra-Rapid (observed half)	150 ps RMS	3–9 hours	15 min
Rapid	75 ps RMS	17–41 hours	15 min
Final	75 ps RMS	12–18 days	15 min

**Fig 1:**
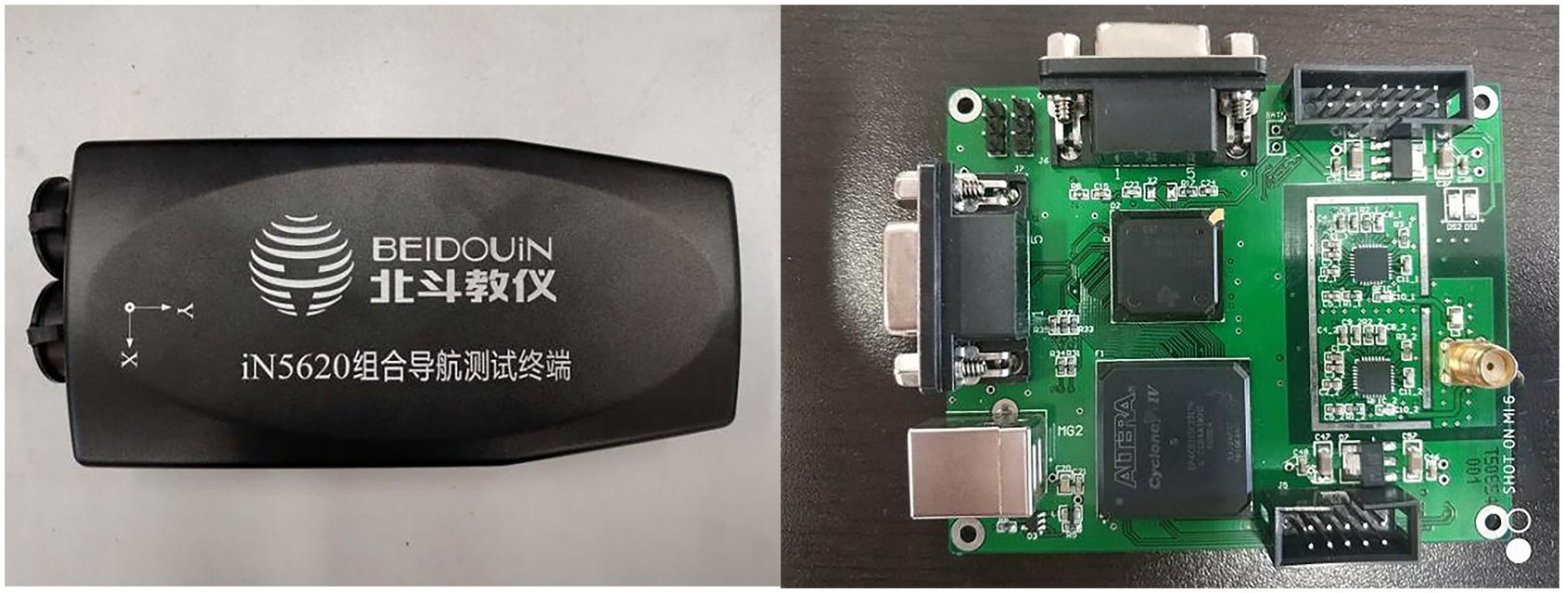
IN5620 navigation test equipment.

The IN5620 navigation test equipment is mainly experimental equipment. And the IN5620 is the main algorithm running platform. Its hardware is mainly composed of RF front-end, baseband processing and other parts. The appearance and circuit board are shown in Fig 1. First download the SCB product from the IGS server (https://cddis.nasa.gov/archive/gps/data/daily/2020/). The sampling interval is 15min. Then read the observation data and ephemeris data provided by the IGS station, and store these data in a certain format, which is convenient for subsequent programs to call the data. Next, at the same time of running different algorithms in the test equipment, observe the experimental results [20].

During the experiment, the IGF is considered to be an accurate SCB with a sampling interval of 15 minutes, which is used to test the proposed SCB prediction model. IGU-P is used as a comparative method to measure the effectiveness of the proposed method. The GPS satellite numbers are listed in Table 2. The SCB sequence of these satellites obtained from IGS is complete and there is no jump. At the same time, we use RMS to evaluate the prediction accuracy to reflect the stability prediction results.

**Table 2:** GPS PRN number.

PRN number
IIR-M 5
IIA 8
IIR 28
IIR-M 31

This paper analyzes and compares the IGU-P, WNN, and Prophet models. The predicted value of the Prophet model is determined according to IGU-O. The three methods respectively predict SCB sequence of the next 24 hours, totaling 96 epochs. Then, compare with the IGF results.

**Fig 2:**
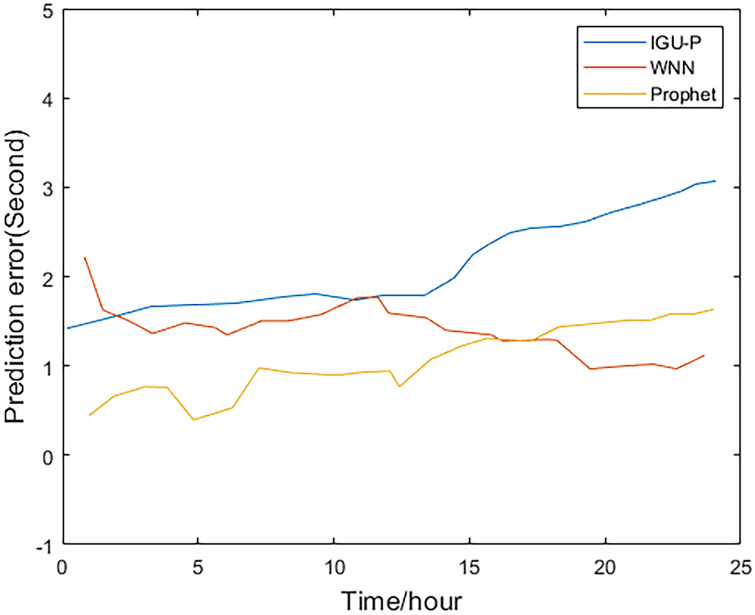
Prediction residuals for PRN 05.

**Fig 3:**
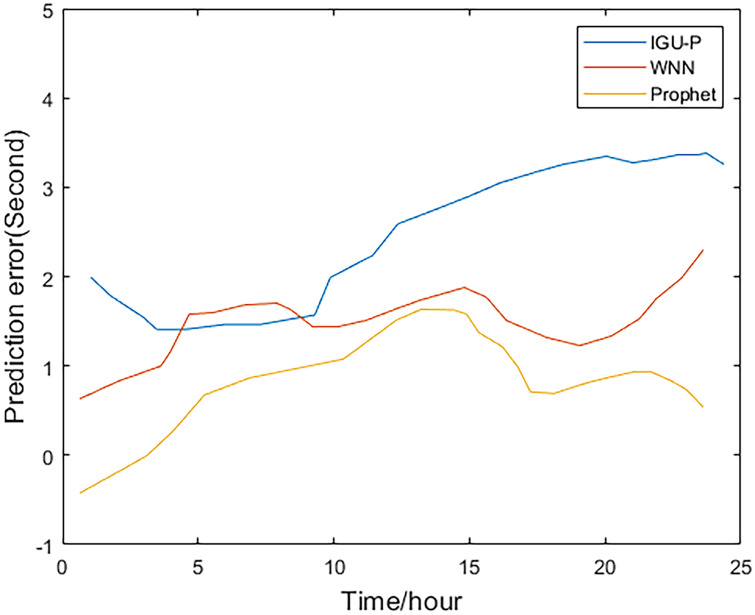
Prediction residuals for PRN 08.

**Fig 4:**
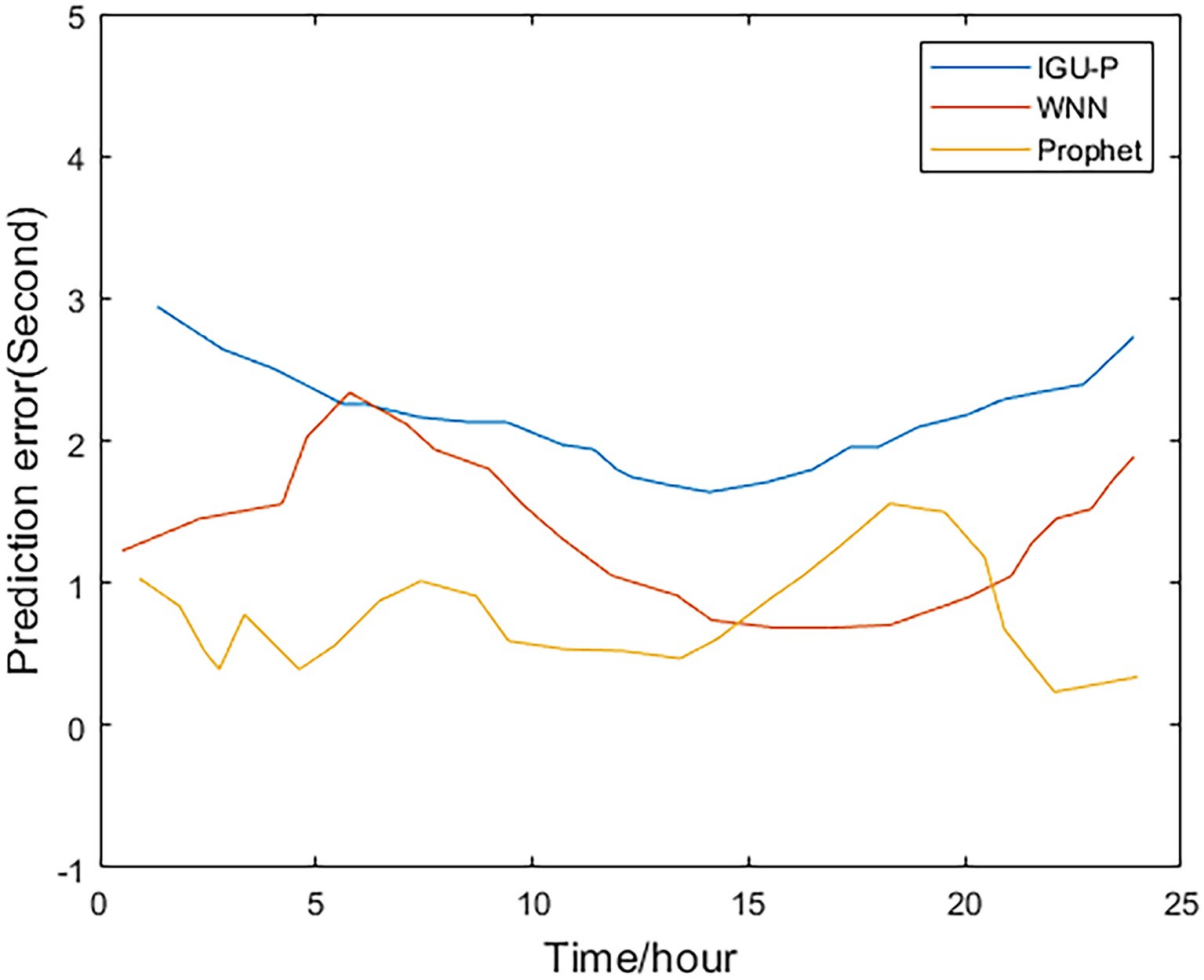
Prediction residuals for PRN 28.

**Fig 5:**
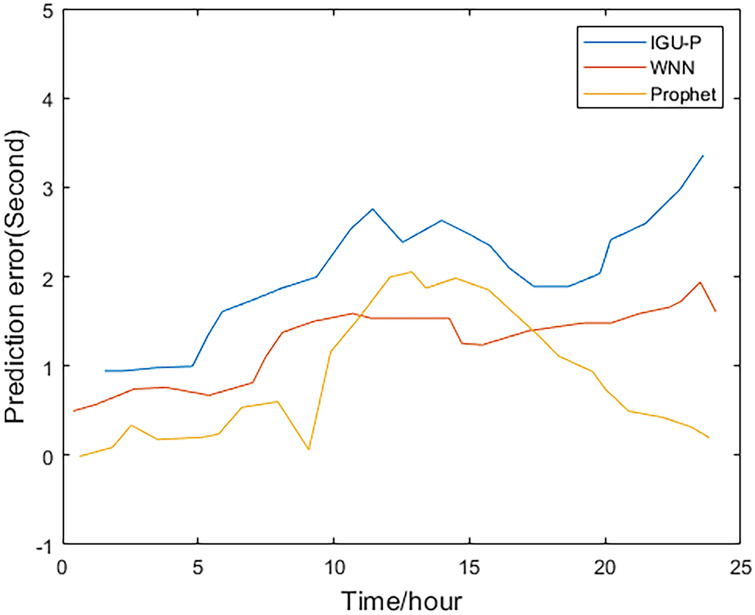
Prediction residuals for PRN 31.

Method one is IGU-P, method two is based on WNN, and method three is the Prophet model. It can be easily seen from Figs 2–5, that compared to SCB given by IGU-P and WNN, the prediction residual of the Prophet model is more concentrated. Specifically, for the RMS average of the four satellites in 24 hours, IGU-P is concentrated around 2ns, WNN is concentrated around 1.5ns, and Prophet model is concentrated around 1ns. This shows that the use of prophet model can significantly increase the accuracy of SCB prediction.

Detailed data is shown in Table 3. In terms of the accuracy of its prediction results, for the selected four satellites, the model proposed in this paper is superior to SCB given by IGU-P and WNN. Specifically, compared with the results of IGU-P, the accuracy of the model in this paper is improved by about 50.3%, 61.7%, 60.4%, and 48.8%; compared with the results of the WNN method, the accuracy is improved by about 21.8%, 35.9%, 40.7%, and 19%.

**Table 3:** RMS values of the three methods(ns).

	Method 1	Method 2	Method 3
IIR-M Rb clock 5	2.2696	1.4405	1.1262
IIA Rb clock 8	2.5945	1.5490	0.9929
IIR Rb clock 28	2.1625	1.4427	0.8555
IIR-M Rb clock 31	2.1390	1.3509	1.0939

**Fig 6:**
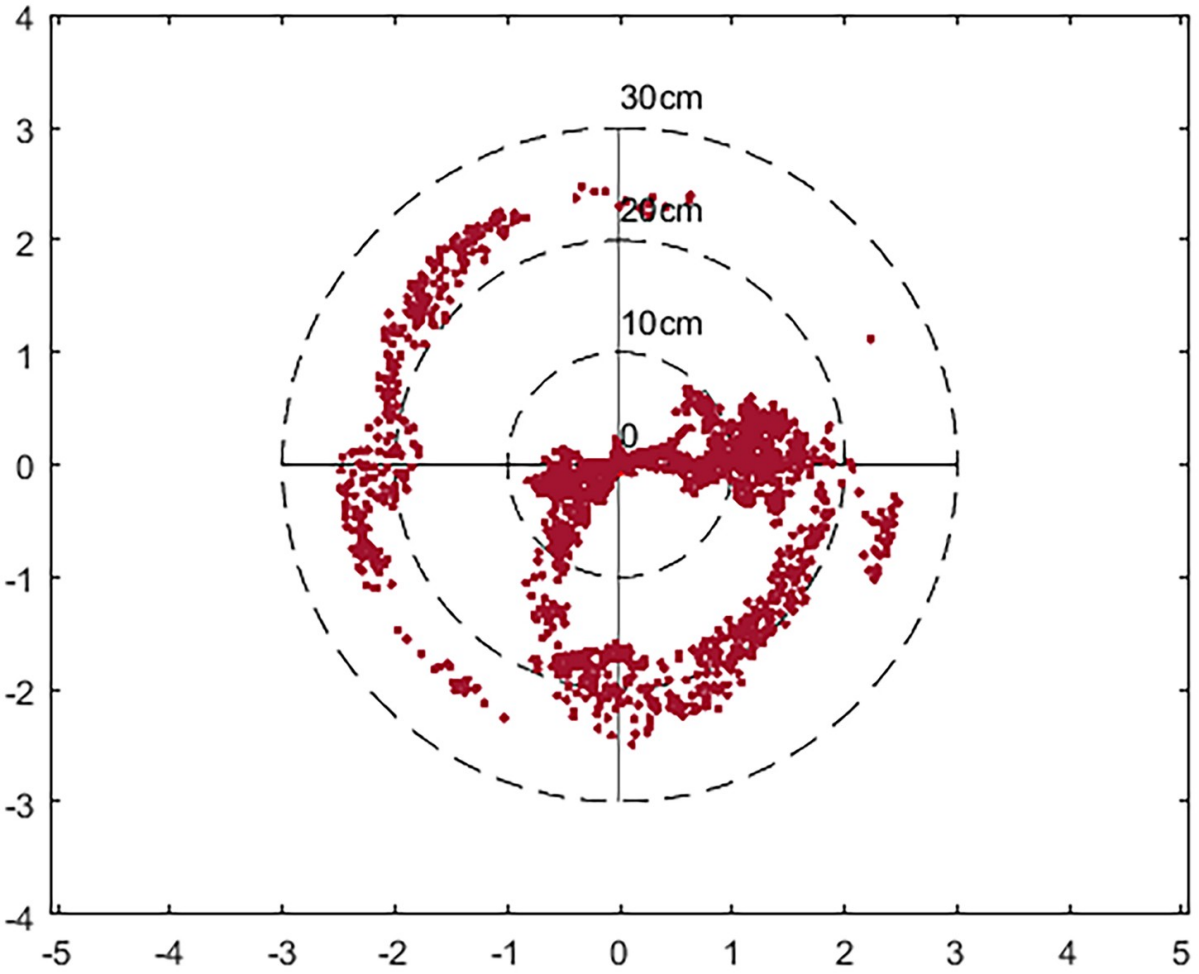
Location distribution map after using IGU-P.

**Fig 7:**
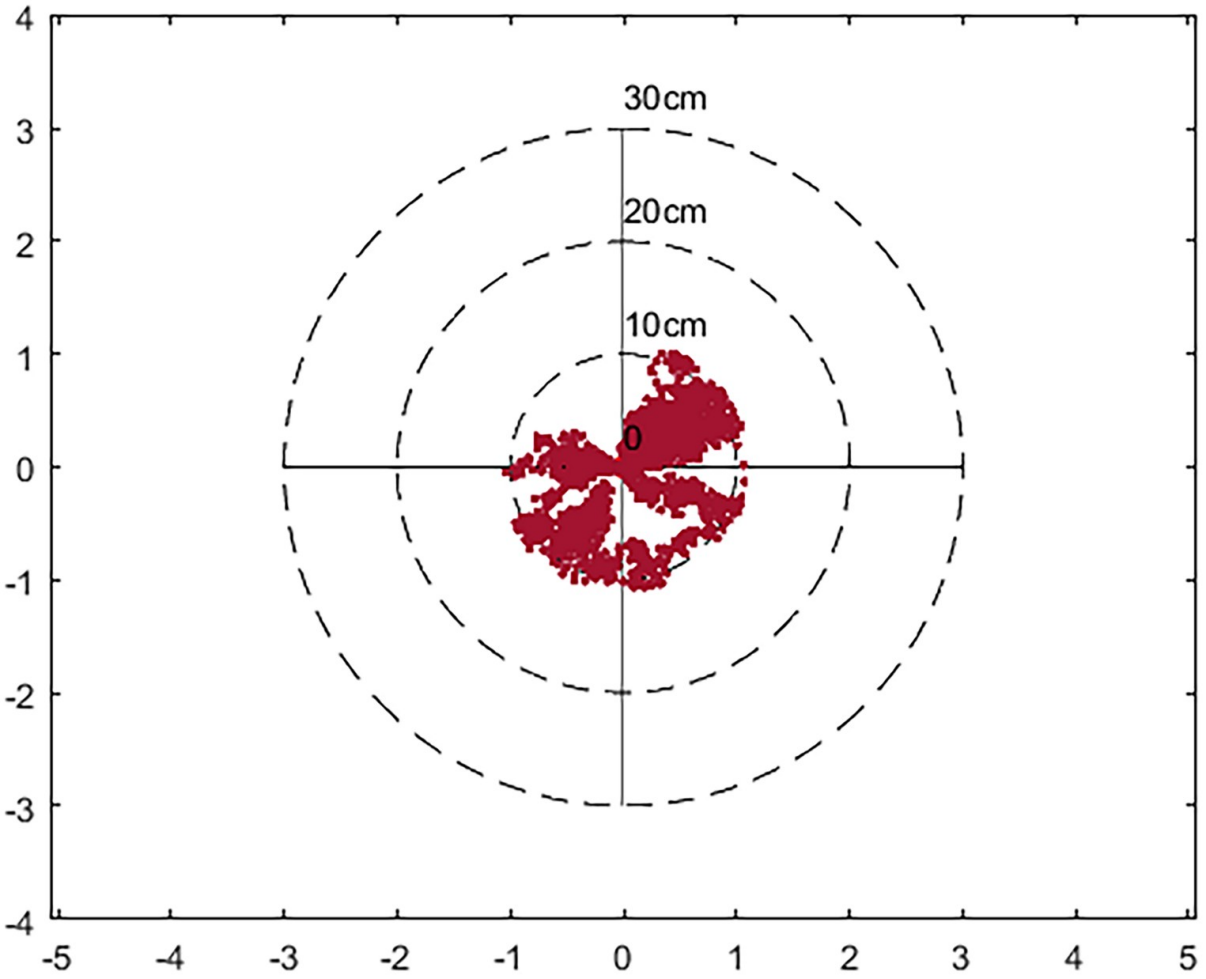
Location distribution map after using Prophet.

Figs 6 and 7 are the location map obtained after using the IGU-P and Prophet models respectively. The zero point is the actual position and the red dot represents the result of the algorithm. It can be seen that the results of using the Prophet model are more concentrated. In terms of the degree of error, the results of IGU-P are concentrated between 0-25cm, and the results of Prophet models are concentrated between 0-11cm. Obviously the latter is more accurate.

**Fig 8:**
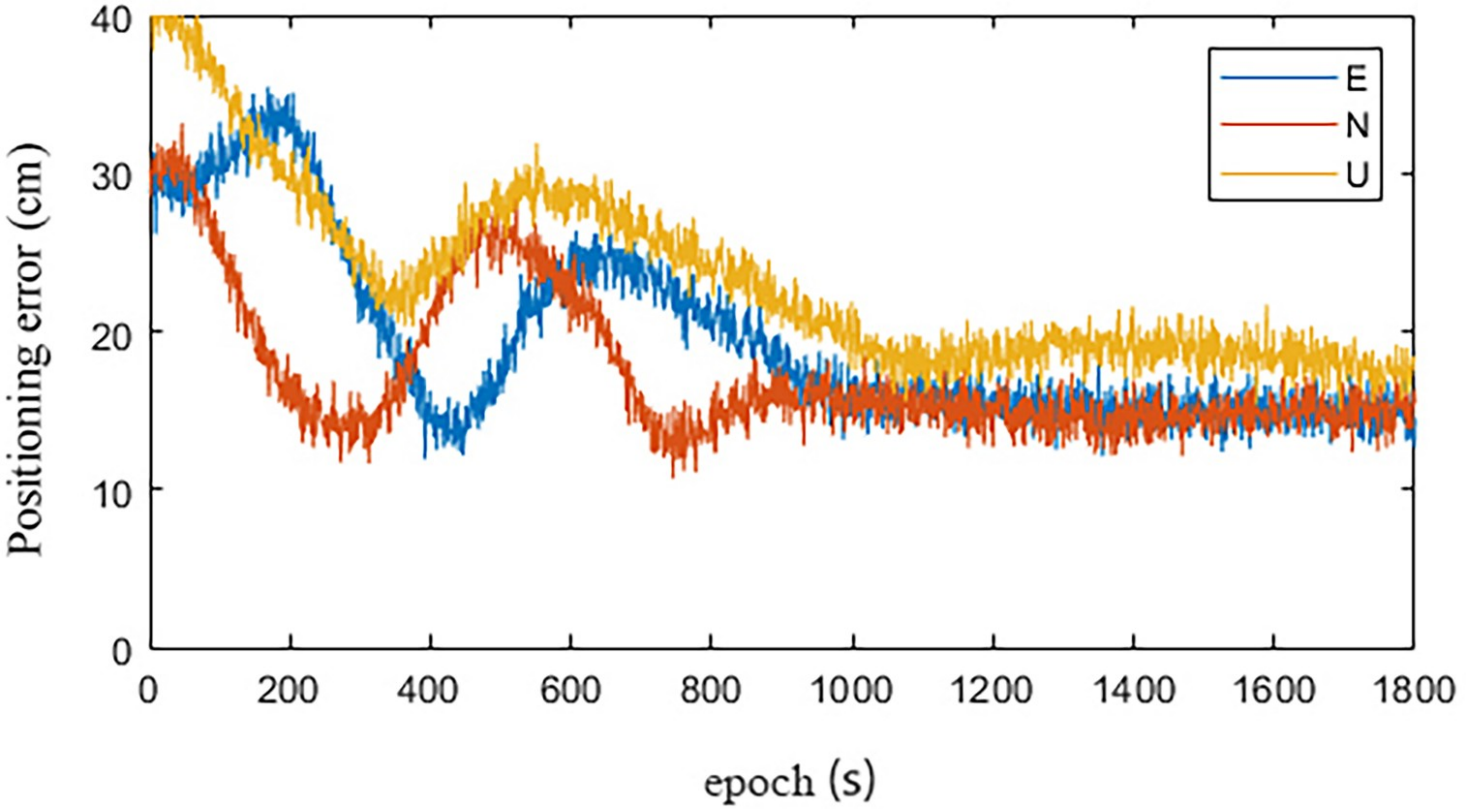
IGU-P.

**Fig 9:**
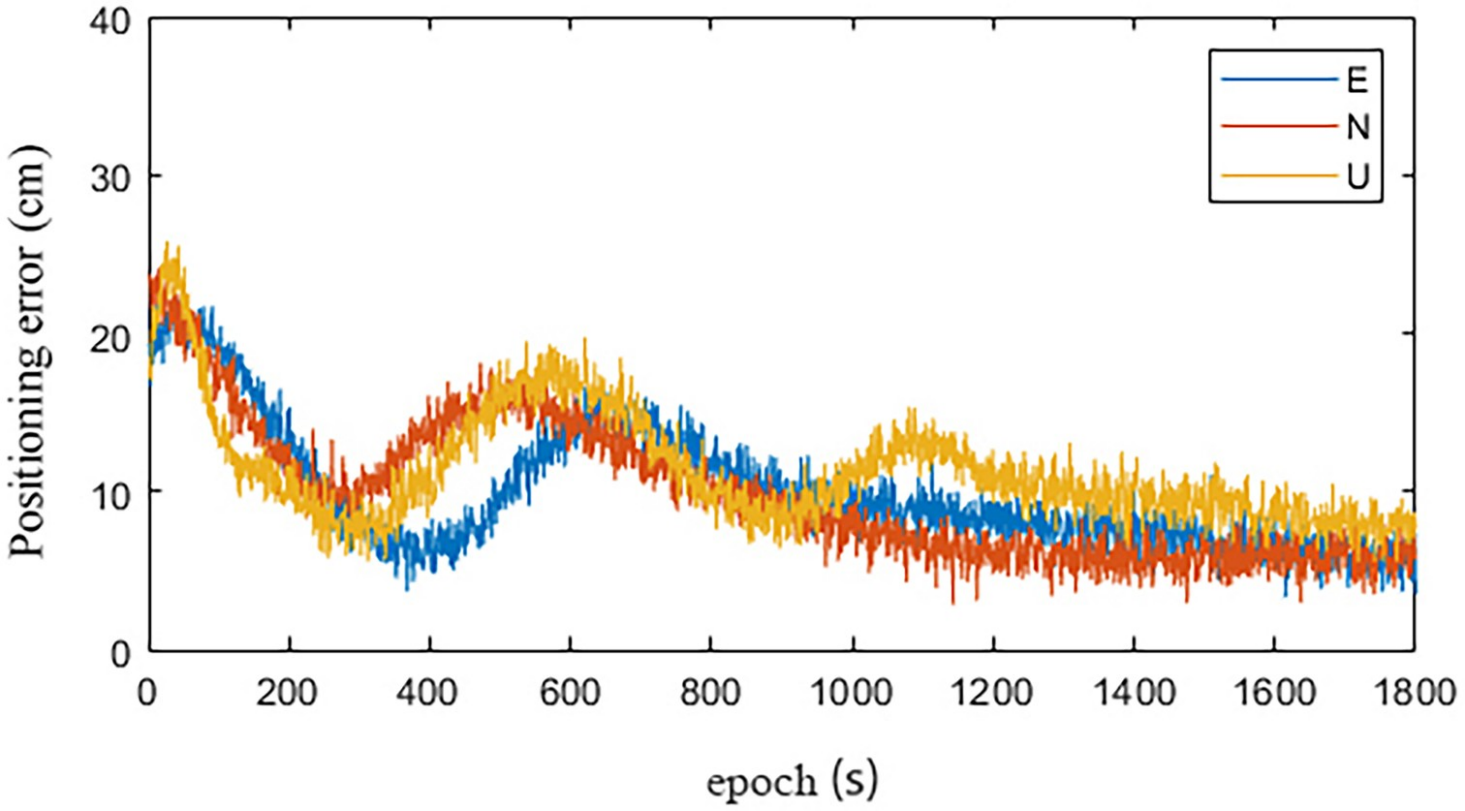
Prophet.

Figs 8 and 9 are the statistical results of the coordinate components of the IGU-P and Prophet models, respectively. NEU is a coordinate system. N is for North, E is for East, U is for Up. It can be seen that standard deviation of each coordinate component is decimeter level. The accuracy in the UP direction is low. In addition to the cause of SCB, other important reasons are that the zenith tropospheric delay model is simple and the estimation accuracy is not high. Obviously, the positioning results have increased by 26%, 35%, and 19% in the N, E, and U directions, respectively.

## 4 Conclusions

This paper proposes a method to improve the accuracy of predicting SCB with Prophet model, which is characterized by trend term, periodic term, random term and error term of SCB. This method first removes the outliers in SCB sequence and then uses the Prophet model to predict SCB sequence. This paper uses SCB of different GPS satellites to analyze the prediction performance of the model, which shows that this method only needs to use less SCB sequence to model SCB. The results verify the effectiveness and feasibility of this method for high-precision short and medium-term GPS SCB prediction. For the selected four satellites, compared with the results of the IGU-P, the accuracy of SCB prediction of the model in this paper is improved by about 50.3%, 61.7%, 60.4%, and 48.8%. In terms of PPP positioning results, this paper uses RTK measurements as a benchmark. Positioning accuracy has increased by 26%, 35%, and 19% in the N, E, and U directions, respectively.

Admittedly, the prediction model used in this paper may not be the best due to theoretical limitations. The next work to be done is to further study the characteristics of satellite clock bias and analyze the corresponding data characteristics, so as to build a more optimized satellite clock bias prediction model and better improve the prediction accuracy and stability.
